# Which is the best ^9^Be carrier for ^10^Be/^9^Be accelerator mass spectrometry?

**DOI:** 10.1016/j.mex.2021.101486

**Published:** 2021-08-11

**Authors:** Silke Merchel, Régis Braucher, Johannes Lachner, Georg Rugel

**Affiliations:** a Helmholtz-Zentrum Dresden-Rossendorf, Dresden, Germany; b Isotope Physics, University of Vienna, Austria; c CEREGE, CNRS, Collège de France, IRD, INRAE, Aix Marseille Univ., Plateau de l'Arbois, Aix-en-Provence 13545, France

**Keywords:** AMS, Phenakite, Beryl, Carrier, Blank

## Abstract

Commercial ^9^Be solutions used for chemical preparation of samples for accelerator mass spectrometry contain the cosmogenic long-lived radionuclide ^10^Be at elevated but different ^10^Be/^9^Be levels. Within a systematic study of recently produced solutions, comparison to published data and new data on customised solutions from minerals, we recommend - if no customised solution is available - the ^9^Be solutions from Australian Chemical Reagents (ACR) or from LGC. They contain ^10^Be/^9^Be at the 3.4 × 10^−15^ level, which is still suitable for the majority of Earth science applications, compared to customised solutions at the 10^−16^ level for lowest-level studies. Commercial solutions from Scharlab having different lot numbers, i.e. an identification number assigned to a particular lot of material from a single manufacturer, vary in ^10^Be/^9^Be by up to a factor of nine. Hence, it seems an advisable strategy to buy a bigger quantity of a single production batch (such as 10 × 100 ml bottles of ^9^Be at 1 g l^−1^) and have them tested once at any AMS facility before first use.

• The best ^9^Be carrier for low-level ^10^Be/^9^Be applications is a customised one from minerals like phenakite.
• The best ^9^Be carriers for medium- and high-level ^10^Be/^9^Be applications are currently from Australian Chemical Reagents (ACR) or from LGC.
• As ^9^Be carriers from Scharlab of different batches (LOT) contain ^10^Be/^9^Be at different levels, it is advisable to buy a bigger number of bottles of the same LOT of commercial carriers after being identified to have reasonably low isotope ratios.

Specifications table

**Table:** 

Subject Area:	Earth and Planetary Sciences
More specific subject area:	*Cosmogenic nuclide*
Method name:	*Best-suited ^9^Be carrier*
Name and reference of original method:	*Best-suited ^9^Be carrier (described in Merchel et al., 2008; doi:10.1016/j.nimb.2008.07.03)*
Resource availability:	*not applicable*

## Method details

### Background and motivation

The technical development of accelerator mass spectrometry (AMS) within the last 30 years has led to at least one order of magnitude lower detection limits for cosmogenic radionuclides such as ^10^Be [1]. The question if the better AMS technique has opened-up new applications in Earth and environmental sciences or if the technical improvement has been demanded by those users from interdisciplinary research remains unanswered. Very likely, it is a win-win-situation for both communities. However, as chemical preparation of samples is a mandatory step before AMS can be performed, most demanding requirements for lowest detection limits are also essential here.

A key issue for low ^10^Be background from sample preparation is the use of a ^9^Be carrier, usually in the form of acidic solution, which contains “no” intrinsic ^10^Be. It has been noted as early as 1984 [2] that commercially available ^9^Be compounds can contain ^10^Be/^9^Be at the 10^−14^ level making them not well-suitable for applications like surface exposure dating. As well-shielded – from cosmic rays – beryllium minerals like beryl were measured lower as 1.7 × 10^−15^ [2], experienced scientists have therefore produced their own ^9^Be carrier solution from those minerals (e.g., [3], [4], [5]). Only this way samples with lowest ^10^Be concentrations could have been successfully measured above background and led to exceptional research work such as the reconstruction of glacier changes by surface exposure or - combined with ^26^Al data - burial dating (e.g., [[6], [7], [8], [9], [10]–11]). Two more examples are the ^10^Be dating of a boulder from a medieval rock fall triggered by an earthquake [12] and the determination of stellar and thermal neutron capture cross sections of ^9^Be [13]. In the past, there had been a few exceptions for commercial carriers being also acceptably low, e.g., one from the company MERCK used for more than twenty years at the now-closed AMS facility at Gif-sur-Yvette [14] and those from the company Scharlab being for a certain production time in the 10^−16^ and 1-2 × 10^−15 10^Be/^9^Be range, respectively [3]. As a newer (since 2006) MERCK solution and also all tested solutions from five other companies were measured to 8 × 10^−14^ to 4 × 10^−14^ (Fig. 1 in [3]), either customised carriers from minerals (beryl, phenakite) or Scharlab carriers have been predominantly used for projects in Earth and environmental sciences.

Besides the enormous effort including working with large amounts of carcinogenic Be powder and extremely toxic hydrofluoric acid, the drawback of customised carriers is the need for accurate ^9^Be determination in the produced solution. Earlier work [3] has shown that the ^9^Be concentration could be determined by three different methods – graphite-furnace atomic absorption spectrometry (GF-AAS), flame-AAS and gravimetry – as precise as 0.3%, which has to be added to the overall uncertainty of all ^10^Be AMS data. Unexpectedly, later work [4] collected results from inductively coupled plasma mass spectrometry (ICP-MS) and optical emission spectrometry (ICP-OES) differing at the 8% level in a first attempt, resulting in an overall uncertainty of 0.5% for the ^9^Be concentration only after remeasurements and additional laboratories being involved in a kind-of round-robin exercise. Hence, customised ^9^Be carrier solutions might add an additional (higher) uncertainty or even a source of systematic bias to ^10^Be AMS data, when single-lab (precise) data, but not several-lab accurate data are taken-into account.

**Fig. 1:**
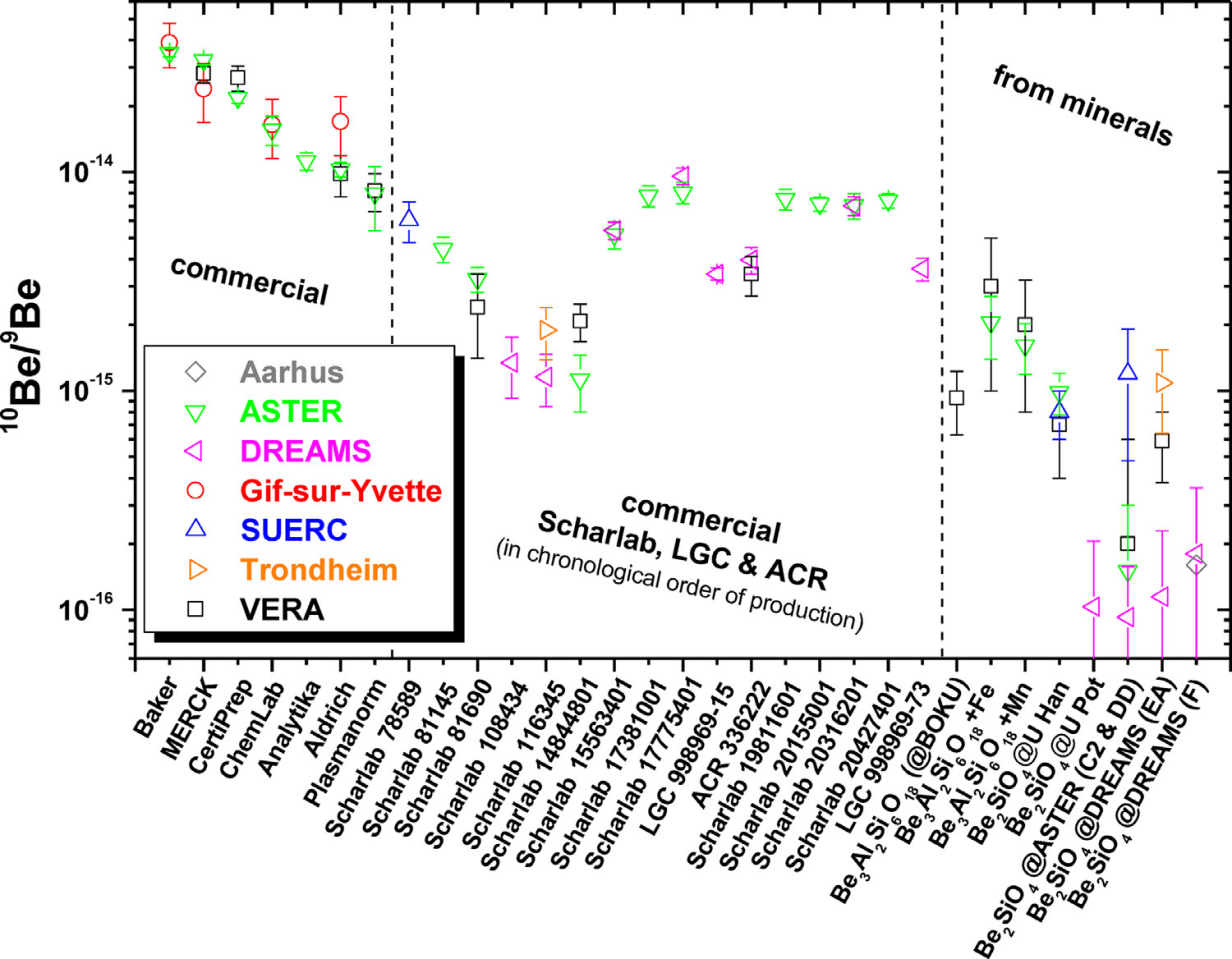
^10^Be/^9^Be results of different commercial and customised (from minerals) ^9^Be carrier solutions measured at seven European AMS facilities. All results from Gif-sur-Yvette and SUERC, and results for Baker, MERCK, CertiPrep, ChemLab, Aldrich, Plasmanorm, and Scharlab with lower LOT numbers than 100,000 have been taken from Merchel et al. [3], thus production dates of solutions are 2007 and earlier. The commercial solution from Analytika is from 2019. Data from Aarhus (no uncertainty given) is from Heinemeier et al. [15]. Carriers made from phenakite, i.e. Be_2_SiO_4_, “@U Pot”, “@U Han”, “@DREAMS (F)” have been produced at the university of Potsdam in 2016, the university of Hanover and at the DREAMS laboratory in 2014, respectively (all unpublished); carrier from beryll, i.e. Be_3_Al_2_Si_6_O_18_ (@BOKU) has been produced at the University of Natural Resources and Life Sciences, Vienna. All other customised carriers have been described in Merchel et al. [3,4]. Values of commercial solutions (left) and customised from minerals (right) have been ordered in descending ^10^Be/^9^Be values, whereas “commercial Scharlab, LGC & ACR” has been ordered according to the production date of the solutions. Uncertainties depicted are on the 1 sigma-level and represent Poisson counting statistics as well as the scatter of multiple measurements on targets of the same material. Each datapoint corresponds to at least 1 event detected. If measurements on a single cathode at DREAMS yielded 0 events, the overall result was evaluated using the statistical approach of Feldman and Cousins [23].

To conclude, based on word-of-mouth recommendation several scientists have used the Scharlab ^9^Be carrier for their applied research projects in the last decade. Unfortunately, the ^10^Be/^9^Be value of more recently produced Scharlab solutions have shown increased levels of ^10^Be/^9^Be, i.e. from 1 × 10^−15^ for LOT number 14844801, over 5 × 10^−15^ for LOT number 15563401 to finally ≥ 7 × 10^−15^ for LOT numbers 17381001 and higher (Fig. 1), thus reaching blank levels, which are not any more acceptable for certain applications. Therefore, users of AMS facilities have been more and more interested in having their Scharlab or other commercial carriers (Analytika, Australian Chemical Reagents (ACR), LGC) tested before first real use. As AMS measurements of carrier solutions are cost- and time-intensive blocking precious AMS time for research projects, we would like to present here, the current status of ^10^Be/^9^Be determinations for different companies and LOT numbers. This way, we are hoping that we and other AMS facilities can spare some further tests and encourage the user and AMS community to spread word-of-mouth recommendations about their lowest commercial ^9^Be carrier, but also to include important information about the used carrier in future scientific papers.

### AMS details

New ^10^Be/^9^Be data added to the data set presented in [3], are published elsewhere [15] for the 1 MV AMS facility Aarhus while setting-up the facility, or have been investigated due to requests by (external) users. This way the data collection (Fig. 1) combines unpublished data from the following AMS facilities:

• ASTER (Accélérateur pour les Sciences de la Terre, Environnement, Risques), Aix-en-Provence, France [16]
• DREAMS (DREsden AMS), Dresden, Germany [17,18]
• Trondheim, Norway [19]
• VERA (Vienna Environmental Research Accelerator), Vienna, Austria [20].

Compared to their earlier measurements [3], ASTER and VERA have changed their set-ups with respect to an ion-source with higher BeO^−^ currents (ASTER) and improved ^10^Be detection using a passive absorber, consisting of a stack of silicon nitride foils, for isobar suppression (VERA). All values are traceable to the primary NIST SRM 4325 standard with the currently most accepted value of (2.79 ± 0.03) x 10^−11 10^Be/^9^Be [21] via in-house secondary standards. No further blank corrections e.g., by subtracting machine blank values have been applied.

### Conclusion and recommendation

This study is a regionally-influenced snapshot of the last years and we would like to raise awareness that the "best manufacturer" can change over time. However, currently we can recommend the ^9^Be carrier (LOT 998969-15; purchased in 2019) from LGC being as low as (3.42 ± 0.22) x 10^−15 10^Be/^9^Be or the ACR (LOT 336222; purchased in 2019) being as low as (3.41 ± 0.70) x 10^−15 10^Be/^9^Be. Another more recent batch from LGC (LOT 998969-73; purchased in 2021) gave the same result within uncertainty, i.e. (3.61 ± 0.43) x 10^−15^, as the LGC batch from two years earlier. There is no difference within 1-sigma uncertainty between most of the ^10^Be/^9^Be data from the same commercial carrier solutions measured at different AMS facilities showing the excellent comparability (Fig. 1). In reverse, measuring a commercial carrier solution at a single AMS facility will produce AMS data valid also for other facilities. As commercial solutions from the company Scharlab having different LOT numbers, i.e., an identification number assigned to a particular lot of material from a single manufacturer, vary in ^10^Be/^9^Be by up to a factor of nine, we strongly recommend to buy a bigger quantity of a single production batch (such as 10 × 100 ml bottles of ^9^Be at 1 g l^−1^) and have them tested once at any AMS facility before first use.

At the level of customised carriers, which can be as low as 9 × 10^−17^, comparisons of different solutions measured at the same facility will allow to find out the best. However, if the same customised solution is measured at different facilities, the extremely low counting statistics are influenced by the use of standards at different levels, e.g., 10^−11^ vs. 10^−12^, and general long-term-memory of ion sources (e.g., [18]). Ineffective isobar suppression (from boron) should not influence the results at sophisticated facilities, but differences in measurement efficiencies at different facilities might result in higher detection limits when detecting “zero counts”. As chemical processing will also increase the final ratio of the so-called processing blank, there are only rare cases when you have to choose between a customised “<10^−16^“ or a “5 × 10^−16^“ ^10^Be/^9^Be carrier [22]; both carriers at this low level will be suitable for nearly all applications.
